# Factors affecting access to primary health care services for persons with disabilities in rural areas: a “best-fit” framework synthesis

**DOI:** 10.1186/s41256-018-0091-x

**Published:** 2018-12-25

**Authors:** Ebenezer Dassah, Heather Aldersey, Mary Ann McColl, Colleen Davison

**Affiliations:** 1 School of Rehabilitation Therapy, Queen’s University, Louise D. Acton Building, 31 George Street, Kingston, Ontario K7L 3N6 Canada; 2 Department of Public Health Sciences, Queen’s University, Carruthers Hall, 62 Fifth Field Company Lane, Kingston, Ontario K7L 3N6 Canada

**Keywords:** Primary health care, Access, Rural, Disability, Review

## Abstract

**Background:**

Access to primary health care (PHC) is a fundamental human right and central in the performance of health care systems, however persons with disabilities (PWDs) generally experience greater barriers in accessing PHC than the general population. These problems are further exacerbated for those with disabilities in rural areas. Understanding PHC access for PWDs is particularly important as such knowledge can inform policies, clinical practice and future research in rural settings.

**Methods:**

We conducted a synthesis of published literature to explore the factors affecting access to PHC for PWDs in rural areas globally. Using an adapted keyword search string we searched five databases (CINAHL, EMBASE, Global Health, Medline and Web of Science), key journals and the reference lists of included articles. We imported the articles into NVivo and conducted deductive (framework) analysis by charting the data into a rural PHC access framework. We subsequently conducted inductive (thematic) analysis.

**Results:**

We identified 36 studies that met our inclusion criteria. A majority (*n* = 26) of the studies were conducted in low-and middle-income countries. We found that PWDs were unable to access PHC due to obstacles including the interplay of four major factors; availability, acceptability, geography and affordability. In particular, limited availability of health care facilities and services and perceived low quality of care meant that those in need of health care services frequently had to travel for care. The barrier of geographic distance was worsened by transportation problems. We also observed that where health services were available most people could not afford the cost.

**Conclusion:**

Our synthesis noted that modifying the access framework to incorporate relationships among the barriers might help better conceptualize PHC access challenges and opportunities in rural settings. We also made recommendations for policy development, practice consideration and future research that could lead to more equitable access to health care. Importantly, there is the need for health policies that aim address rural health problems to consider all the dimensions and their interactions. In terms of practice, the review also highlights the need to provide in-service training to health care providers on how to enhance their communication skills with PWDs. Future research should focus on exploring access in geographical contexts with different health care systems, the perspectives of health care providers and how PWDs respond to access problems in rural settings.

**Electronic supplementary material:**

The online version of this article (10.1186/s41256-018-0091-x) contains supplementary material, which is available to authorized users.

## Background

Equitable access to health care is a major principle of national health systems globally [1, 2]. However, persons with disabilities (PWDs) generally experience greater barriers in accessing PHC than the general population, and these problems are further exacerbated for those with disabilities in rural areas [3]. PWDs in rural settings confront a wide range of informational, geographical and financial barriers to health care access [3, 4]. These barriers can lead to negative health outcomes and widen rural health disparities between PWDs and the general population [5]. In the past decade there has been a growing interest in the study of health care access for rural residents, particularly in Australia, Canada and United States, where there is a long tradition in rural health care research. Similar studies have also been conducted recently in low-and middle-income countries (LMICs) [6, 7]. This review seeks to identify and synthesize evidence regarding factors affecting access to primary health care (PHC) for PWDs in rural areas globally.

PHC is an approach that encompasses health policy and service provision that is delivered at the individual level (i.e. primary care services) and population level (public health) [8]. Within the health services delivery domain, PHC is broadly regarded as the first level of contact that health consumers have with the health care system [9]. Care services under PHC may include: health education; environmental health; public health nutrition; reproductive and family health; immunization against common communicable diseases; epidemiological investigation and disease control; appropriate treatment of common ailments and injuries; and provision of essential drugs [10, 11].

We use Russell and colleagues’ [2] conceptual framework for evaluating access to PHC in rural communities, particularly for PWDs, in conceptualizing the review. In this framework, access is conceptualized as the “fit” between the characteristics of the individual/client (i.e. PWD) and the characteristics of the health care system. Access is thus defined as the ease with which PWDs can seek and obtain health services when the need arises [1, 2]. According to Russell and colleagues’ [2] framework, access to PHC is achieved through the following seven dimensions; availability, geography, affordability, accommodation, timeliness, acceptability and awareness.

Most of the existing reviews on disability and health care access to date have been mostly focused on the following PHC services: preventive, screening and oral health for PWDs [12]; water and sanitation for PWDs [13]; oral health care among persons with intellectual and learning disabilities [14, 15]; maternity services for women with physical disabilities [16, 17]; health care access for PWDs who are members of underserved racial/ethnic groups in the United States [18] and persons with hearing impairments [19]. These studies are mostly urban centric and focus mainly on the barriers to health care services for PWDs. Though a review by Lishner and colleagues [3] delved into the perspectives of rural residents with disabilities about access to health care, the authors mainly focused on rural care in the United States, and only examined studies published up to 1996.

Evidence suggests that access to health care and services is the major concern for rural populations globally [20, 21]. Further, researchers have identified access to appropriate health care services as the number one research priority for PWDs [22], including those in rural areas. To date primary empirical studies, with diverse and sometimes contradictory findings, from a wide range of countries have provided insights into PHC access for PWDs in rural areas. Our goal in conducting a synthesis of these studies is to provide a holistic and comprehensive understanding of this wide range of primary research studies.

This review therefore seeks to identify existing evidence regarding factors affecting access to PHC services in rural areas worldwide. A global picture of such evidence is timely as the recent United Nations Declaration on Sustainable Development Goal 3 emphasizes universal health coverage, access to quality health and equity in health care as key to achieving the overall health goal for sustainable development [23]. Furthermore, this review provides insight that is useful in assessing health policies, improving clinical practice and advancing knowledge on PHC access for PWDs in rural areas globally.

## Method

### Review design

The methodological approach for this review is based on framework synthesis [24]. We specifically adopted the “best fit” framework synthesis [25, 26]. The “best fit” approach is a recent development, adapted from framework analysis, which involves systematically organizing data into a prior conceptual framework [25–27]. We used this approach for three reasons. First, there is a prior framework (i.e. rural centred PHC access framework) that can inform sorting and charting of the data. Second, the approach increases coding transparency and fosters teamwork in analysing the data [27]. Finally, although the approach is largely deductive (testing a framework), it also includes inductive (thematic) analysis that is useful in understanding a phenomenon [25, 26], especially rural health access for PWDs. Thus, the “best fit” approach capitalizes on the strengths of both framework synthesis and thematic synthesis [26, 27].

### Search strategy

We comprehensively searched for relevant literature using five electronic databases—CINAHL, EMBASE, Global Health, Medline and Web of Science. The first author in collaboration with a health sciences librarian developed the search strategy. We included all possible key words for three main areas relevant to the review: PWDs, PHC and rural (See details in Table 1). We conducted the search using a combination of medical subject headings (MeSH) key terms and free text adapting the syntax required for each database.

**Table 1:** Detailed search terms

	CINAHL (Via EBSCOhost)	EMBASE (Via Ovid)	Global Health (Via Ovid)	Medline (Via Ovid)	Web of Science
Persons with disabilities	(MH“Disabled+”) OR Disab*	exp disability/OR exp disabled person/OR disab*.mp.	exp disabilities/OR exp people with mental disabilities/OR exp children with disabilities/OR exp people with disabilities/OR exp learning disabilities/OR exp people with physical disabilities/OR disab*.mp	exp Disabled Persons/OR disab*.mp.	Disability
Primary Health Care	(MH “Primary health Care”) OR (MH “Medical Care”) OR (MH “Health Services Accessibility+”)	Exp primary health care/OR exp primary medical care/OR exp “health care cost”/OR exp health care delivery/ OR exp health care quality/OR exp health care access/ OR exp health service/OR exp health care/OR exp health care system/OR exp health care utilization/	exp primary health care/OR (community health OR health care OR health services OR Community health services OR medical services).sh.	exp Primary Health Care/OR exp Healthcare Disparities/OR exp “Delivery of Health Care”/OR exp Health Services Accessibility/OR exp “Health Services Needs and Demand”/	Primary health care
Rural or Remote	(MH “Rural Areas”) OR (MH “Rural Health Personnel”) OR (MH “Rural Health Centers”) OR (MH “Rural Health Services”) OR (MH “Rural Population”) OR (MH “Hospitals, Rural”) OR “rural*” OR “remote health”	exp rural area/ OR exp rural population/exp OR rural health care/OR exp rural urban difference/OR rural*.mp OR remote health.mp. OR	exp rural environment/ or exp rural communities/or exp rural society/or exp rural areas/or exp rural health/ or exp rural settlement/OR exp rural population/OR rural*.mp.	exp Rural Health/OR exp Hospitals, Rural/OR exp Rural Population/ OR exp Rural Health Services/OR exp Telemedicine/OR rural*.mp. OR remote health.mp.	Rural

### Study selection

We exported the search results into Refworks (a reference management software) and selected relevant studies based on the following inclusion/exclusion criteria:

- *Study design* We included quantitative, qualitative and mixed methods primary empirical studies that explored at least one of the dimensions in the rural access framework [2]. We excluded review articles, dissertations/thesis, commentaries, letters to editors, case reports, book reviews and chapters or articles that did not report a primary study.
- *Language, source and time period* We included English language, peer reviewed articles published between 2006 and early November 2017. We chose 2006 as the cut-off point because it was the year in which the United Nations adopted the Convention on the Rights of Persons with Disabilities (CRPD), which guarantees access to health care for PWDs as a fundamental human right [28]. As such, various countries have ratified the CRPD and set out policies that are in line with its principles. We also adopted November 2017 as the end date as it was the month prior to when we conducted the review. We excluded non-English articles because we could not immediately access translation services.
- *Study participants* We included articles that focused on any type of disability (e.g. physical, mental, vision, hearing, intellectual and developmental disabilities). We also included studies that compared PWDs and those without disabilities provided it was feasible to identify and separate the perspectives of those with disabilities. We did not include disabilities associated with HIV/AIDS related. Although this condition is recognized as a form of disability and included in a rural health review [3], the complexity and uniqueness of this population in recent times may require its own study. As a result of this, we excluded all the articles that explored disability and HIV/AIDS access to health care (especially anti-retroviral drugs) or those articles that explored the perspectives of PWDs who also have HIV/AIDS.
- *Phenomenon of interest* We included PHC that relates to primary medical care including: (a) treatment of diseases and injuries; and (b) provision of essential drugs. We included these two primary core services because they are the urgent care needs for minority groups such as PWDs in many rural communities [29]. We define these core services as basic health services/care that health care practitioners, including family physicians and nurses, provide to PWDs especially in rural areas. We excluded studies that focused on access to secondary or tertiary health care.
- *Research setting* We adopted “rural” as defined within each of the article rather than choosing a definition. We took this decision because evidence suggests that there is no universally accepted definition of *rural* [30–33]. We also included studies that involved rural and urban areas provided it was feasible to extract the rural portions of such studies.

### Screening of articles

Two authors independently screened the titles and abstracts of the studies using an exclusion criteria relating to publication type and language, research topic and study population and year of publication. We resolved discrepancies through discussion. We retrieved the full-text articles of the remaining studies and then read and independently screened the full text articles to identify eligible studies. At this stage, we resolved discrepancies through discussion, and if required we involved a third reviewer. We also conducted a manual search of disability, health and rural-related journals—*Disability and Rehabilitation; Disability and Health; Disability, CBR and Inclusive Development; Journal of Rural Health; and Rural and Remote Health*. We also searched the reference lists of eligible papers for additional studies. Finally, we used the titles of all eligible articles on Google Scholar’s “cited by” and “related articles” to identify potential articles.

### Data extraction and synthesis

We imported the included studies into NVivo 11, a software program for managing data. Two reviewers extracted and coded the findings/results sections of the included studies into the seven dimensions of the rural PHC access framework [2]. Table 2 provides the operationalized definitions of each of these dimensions.

**Table 2:** Rural primary health care access framework [2]

Dimensions	Operationalized definitions
1. Availability	Relates to the volume and types of services and facilities in relation to the needs of the clients.
2. Geography	Refers to the proximity of health services or providers to clients, and also the ways that clients’ can transcend the distance between their location and that of the services or providers.
3. Affordability	Relates to clients’ ability to pay the overall costs of health care services, including direct and indirect cost of care.
4. Accommodation	Involves the ways PHC resources are organized in relation to the clients’ ability to contact with, gain entry to and navigate the system.
5. Timeliness	Reflects the extent to which care can be sought, offered or received within a time frame and which is optimal to achieve the best health outcomes.
6. Acceptability	Relates to the attitudes and beliefs of consumers about the health care system to the personal and practice characteristics of health care providers.
7. Awareness	Involves sharing information between health services and clients, and also enhancing clients’ knowledge about the health care system.

We used each dimension of the framework as a theme for deductive analysis. The framework has alternative terms to each of the access dimensions (i.e. the themes). We used those alternative terms that are relevant to the review as sub-themes. We also inductively analyzed the data that did not fit into the themes or sub-themes of the framework. We followed this process in order to generate new themes and/or sub-themes and understand the phenomenon of study (access to PHC for PWDs in rural areas). For instance, through inductive analysis, we found “Operation Hours” as a new sub-theme within the “Accommodation” theme. This process has recently been successfully used in similar reviews [34, 35].

## Results

### Search results

**Fig. 1:**
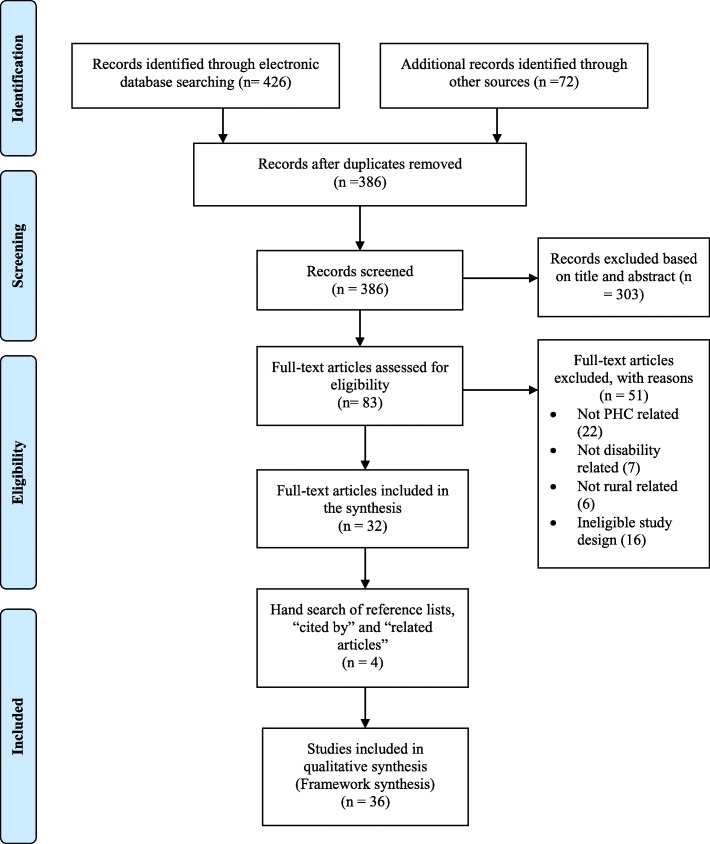
PRISMA Flow Diagram

We screened 386 records after the removal of duplicates from the databases and hand search of key journals. Of the 386 records, we selected 83 full-text articles based on title and abstract. We further screened the 83 articles by reading the full text and reducing the number to 32 relevant articles based on the inclusion/criteria outlined earlier. We then searched the reference lists of the remaining 32 articles, and also used the titles of the articles to search on Google Scholar features “cited by” and “related articles”. This led to the identification of 4 additional articles that met our inclusion criteria for a total of 36 empirical articles. The flowchart summary of literature search is presented in the PRISMA diagram (Fig. 1) [36].

### Characteristics of included studies

Most of the articles (*n* = 33) in this synthesis were published in the last five years 2012–2017, thus indicating a recent interest on this topic. Of the 36 studies, 10 were conducted in high income countries, including Australia [37–40], United States [41–44] and Canada [45, 46]. The remaining 26 studies originated from LMICs primarily from Ethiopia [47–54], South Africa [55–59], India [60, 61], Nepal [62, 63], Malawi [64], Mexico [65], Namibia [66], Pakistan [67], Tanzania [68], Thailand [69, 70] and Vietnam [71]. One article reported studies from four different African countries—Malawi, Namibia, South Africa and Sudan [72].

Most of the studies (*n* = 30) employed qualitative design, four were quantitative and the remaining two were mixed method design. While most of the qualitative studies adopted generic qualitative approaches, five employed specific qualitative traditions including phenomenology [46, 54], grounded theory [55], ethnography [65] and participatory action research [71]. The 4 quantitative articles were cross-sectional studies [44, 59, 69] and a population-based household survey [72]. Twelve of the studies were aimed at rural health care access for PWDs in general. The remaining studies focused on specific disabilities such as physical (*n* = 12), mental (*n* = 7), and intellectual and developmental (*n* = 5). Research participants were mostly adults aged 18 years and above, and included PWDs and their carers (support workers and family members), health care providers (mainstream health practitioners, traditional and faith healers), community members/leaders and policy makers. The sample size of the studies ranged from one participant to as high as 9307 participants. Interviews and focus group discussions were the main data collection sources, while content, framework, thematic analysis, descriptive and inferential statistics constituted the data analysis approaches. (See Additional file 1 for detailed description of the included articles).

### Synthesis of findings

We presented the findings deductively using the seven dimensions as the main themes. The sub-themes we found through inductive analysis are embedded within each of the dimensions (or themes). We also organized the findings in each of the themes.

### Availability

Of the 36 papers, 23 of them addressed availability as a factor affecting access to health services in rural areas. The sub-theme was resources.

#### Resources

The papers highlighted that availability of resources are critical to health care access. This sub-theme has three facets: human resource, health care infrastructure and health services. First, with regard to human resource, the articles noted that health care delivery was hampered by the lack of health care providers [37, 39, 48, 53, 64, 66, 67]. For instance, a study indicated that PWDs in rural Malawi were turned away in health facilities because they were no health care personnel to attend to their health conditions [64]. A paper noted that the limited number of providers in rural areas was sometimes attributed to the difficulties in recruiting personnel due to low salaries [48]. Papers also reported that frequent turnover of staff was experienced in rural communities [39, 48]. Second, some of the papers highlighted that lack of health infrastructure like drugstores and laboratories as well as limited health centers hindered health access [49, 65]. Third, limited supply of drugs and medical equipment were concerns reported in the papers [58, 59, 64–66, 68, 72].

The papers also stressed the importance of resource availability to clients [47, 58]. One paper particularly indicated that making mental health services available in a community can enhance the quality of life, functioning and productivity of people with severe mental disorders [47].

### Geography

Twenty-eight of the 36 papers addressed how geography determined health care access. Within this theme, we identified two sub-themes, and these were distance and transportation to a facility, and terrain and climate.

#### Distance and transportation to a facility

The proximity of clients to health care facilities was highlighted in the papers as a major concern. Articles specifically reported that due to resource constraints, most health care facilities were located in urban areas [45, 53, 62]. Given this, many articles reported that clients had to travel long distances to reach a facility. In addition to distance, the poor nature of roads in most rural areas was highlighted in some of the papers [39, 55, 57–60, 72]. These road networks especially posed a major challenge in travelling to access health care services [60].

Given the location of facilities, the articles also highlighted different modes of transportation that clients used to reach health care service centers. In some of the studies participants discussed walking long distance to reach a health care facility [50, 51, 56, 57, 63, 66, 69]. The use of a wheelchair was the major mode of transportation for those with physical disabilities in a few studies [42, 43, 57, 62, 67, 69]. For instance, a paper reporting on a study in rural Thailand indicated that about 57% (*n* = 462) of people with mobility impairments use wheelchair to reach to a health care facility [69]. A paper in South Africa also demonstrated that in one instance, a parent used a wheelbarrow to transport their son with intellectual and physical disability [56].

The articles also stressed that the provision of public transportation is paramount to health care access [41, 42, 45, 57–59, 62, 65]. Despite this, some of the papers noted that limited public transportation hampered clients’ access to health centers and pharmacies [41, 42, 45, 58, 65]. For instance one article recounted that 16% (*n* = 322) of their study participants with disabilities experienced lack of transport to reach health care facilities [59]. Limited ambulance services also compounded health access challenges in some rural communities [55, 56]. As a result, some papers elaborated how clients have to book transport in advance or pay for private transport services in order to access health [41, 42, 57, 58, 66].

#### Terrain and climate

Given the long distance and limited transportation, papers also recounted the experiences of participants in navigating geographical features as they try to seek care. In particular, persons using wheelchairs in rural South Africa had to navigate mud and gravel [57]. This situation was exacerbated during the rainy season when people had to use their wheelchairs in wet conditions in hilly areas to a facility [62]. Additionally, heavy rains and floods in rural Thailand serve as obstacles to health care providers in providing services to PWDs [70]. Papers also reported that participants encountered rivers, forests, mountains hills and valleys that posed barriers [51, 56, 57, 62]. In one extreme instance, authors noted that people have drowned in water bodies as they attempt to seek care [57].

### Affordability

Of the 36 papers, 27 of them focussed on affordability as a factor affecting health care access. In this theme we noted two sub-themes which were cost of service and indirect cost of care.

#### Cost of medical service

The provision of affordable health care is critical to clients. More particularly, providers in some of the articles noted that the provision of low cost or free health services will ensure equitable access. However, the papers raised concerns about the high cost of medical drugs and other services to clients [48, 66, 68, 72]. Some papers reported that due to poverty among individuals with disabilities, they could not afford drugs and other medical services [43, 54, 67, 68]. In order to address the high cost of care, a few of the papers suggested policy strategies such as health insurance schemes [43, 48] and disability grants [56, 58]. Although insurance schemes potentially subsidize cost, in some instances, clients said their coverage has limitations including insurance companies deciding what should be covered [42, 43].

#### Indirect cost of care

The papers also reported associated cost to the individuals in seeking care. The cost of transportation to obtain health care was particularly noted in some of the papers. In Ethiopia, although medication for podoconiosis was free, two papers elaborated that cost of transportation deterred people from seeking care [50, 51]. One article also indicated that 11% (*n* = 322) of their study participants with disabilities could not afford the cost of transportation to reach health care facilities [59]. Interestingly, some articles reported that participants had to pay extra cost for their wheelchairs and accompanied caregivers [56, 57]. Other associated costs reported in the retrieved articles included accommodation and meals for the duration of seeking care in a nearby facility [47, 51].

### Accommodation

There were 13 of the 36 papers that addressed accommodation as a determinant of health access. The sub-themes were operation hours and architectural designs.

#### Operation hours

A few of the papers discussed the importance of hours of operation of health care facilities in accessing health care services [45, 46, 58, 66]. A study in South Africa reported that most public health care services in rural areas only operated 5 days a week commencing from 7:30 am until 4:30 pm [58]. These hours could not therefore accommodate the needs with those who rely on others to access health care facilities [66]. In view of the operation hours, emergency services outside of these operation days and hours had to be taken to the nearest health centre that was far away. In addition to operation hours, the flexibility or ability of health care providers to forgo some of the bureaucratic procedure was as paramount in ensuring health care services for persons with traumatic spinal cord injuries [46].

#### Architectural designs

This sub-theme focussed on the designs of health care facilities and transport services. Many articles reported that this was especially important for persons with mobility impairments. Some of the papers discussed the arrangement of health care facilities that could not accommodate persons with physical disabilities [43, 44, 46, 57–59, 62, 66, 67]. In particular, the lack of ramps at entrances hampered physical access to health facilities. Even when persons with physical disabilities were able to navigate these physical features, barriers in accessing exam tables, consulting rooms and washrooms within health care facilities were reported [43, 44, 57, 67].

### Timeliness

Thirteen of the 36 papers addressed timeliness as a factor affecting health care access. The sub-themes focussed on wait time to deliver care and consequences of wait time.

#### Wait time to deliver care

The papers identified the time frame that care can be provided to clients as an important determinant of health care access. There were conflicting reports on time in receiving health care. For instance, two studies noted that preferential treatment was offered to clients with disabilities at health care facilities [58, 64]. In some studies authors noted that health care providers specifically served clients with disabilities before others, regardless of their position in a queue. In some of the studies however, timely access to care was reported as a major challenge [37, 38, 46, 57]. One paper particularly highlighted that waiting time can take over half a day on average [57].

#### Wait time consequences

The papers also reported the consequences of timely access to care. One study noted that timely access to treatment for persons with mental disorder will yield better health outcomes and consequently reduce stigma [48]. However, some of the papers indicated that delays in receiving care can increase clients’ risk of secondary conditions [58, 67]. One other study also reported negative consequences of wait time to the individual client and colleagues in a health care facility including fatigue [67].

### Acceptability

Twenty-six (26) of the 36 papers addressed acceptability as a major determinant of health care access. This theme has two sub-themes which were attitudes of health care providers and perceived quality of care.

#### Attitudes of health care providers

A majority of the studies revealed both positive and negative attitudes that affect health care access among PWDs. On the positive side, papers indicated that providers were kind, helpful and willing to treat their clients’ health conditions [58, 61, 63, 64, 66]. At the same time, some providers built a strong relationship/rapport with their clients that supported quality health care delivery [37, 43]. These positive attitudes were partly due to rigorous campaigns in strengthening providers’ attentiveness in meeting the health needs of clients with disabilities [64]. Nevertheless, some of the studies indicated that negative attitudes, including discrimination and stigmatization from providers posed a major barrier in health care access [48, 57, 65–67]. For instance, a paper recounted how providers did not usually provide the same level of care as they would to non-disabled clients [67]. Other papers noted that discrimination emanated as a result of cultural differences between users and health professionals [65, 66]. One article reported that clients’ low self-esteem prevented the provision of appropriate care, this is because clients refused to speak or explain their health conditions to health care providers in Nepal [62].

#### Perceived quality of care

Clients’ perceptions about the care was discussed in some papers. For persons with mental disorders, their decisions to seek care were largely influenced by those with previous experiences at health facilities [47]. In general, clients expressed low satisfaction with care at facilities in the papers. In particular, some clients complained that they stopped receiving care at health facilities due to lack of improvements in their health conditions [50, 65]. Due to the perceived low quality of services some clients resorted to alternative care, including traditional and faith-based healers [47, 49, 50, 54]. Interestingly, a paper stressed that when patients exhausted traditional treatments, Western medical clinics became their last resort [52]. One article also reported that others also rely on medical shops or travel to major cities to seek care [62].

### Awareness

Twenty-three (23) of the 36 articles addressed awareness as a factor affecting health care access. Within this theme, we identified two sub-themes which were knowledge and information and communication.

#### Knowledge

Some of the papers stressed that clients’ and carers’ limited knowledge about services impeded access. For instance, one paper recounted that community members could not recognize people with a mental disorder [55]. Additionally, some articles reported that health care providers and policy makers’ knowledge about services is critical in making services accessible. However, in some cases the articles reported that providers and policy makers exhibited limited knowledge about services [48]. Providers’ lack of knowledge about diagnoses and treatment of disability related health problems was another concern raised in papers [43, 65, 67]. On the contrary, one article reported that 66% (*n* = 142) of study participants with spinal cord injuries indicated that health care providers were knowledgeable about their health conditions [44].

#### Information and communication

Given clients’ limited knowledge, the papers recognized that the provision of information about services could promote health care access. Relatedly, some articles stated that providers’ ability to communicate the kind of health care services readily available can lead to effective health care delivery [37, 39, 43, 58, 61, 66, 71]. Nevertheless, in some cases, health care providers had difficulties in communicating with clients with intellectual and hearing impairments [37, 38, 62, 66]. The inability of providers to comprehend the level of understanding of clients with intellectual impairments was raised in a paper as a barrier to health care delivery [37]. Another paper indicated that providers could not also convey information to or communicate in sign language with persons with hearing impairments [66]. As a result of this, two papers recounted that providers relied on carers to report clients’ health conditions [37, 62].

### Linkages of the health care access dimensions

**Fig. 2:**
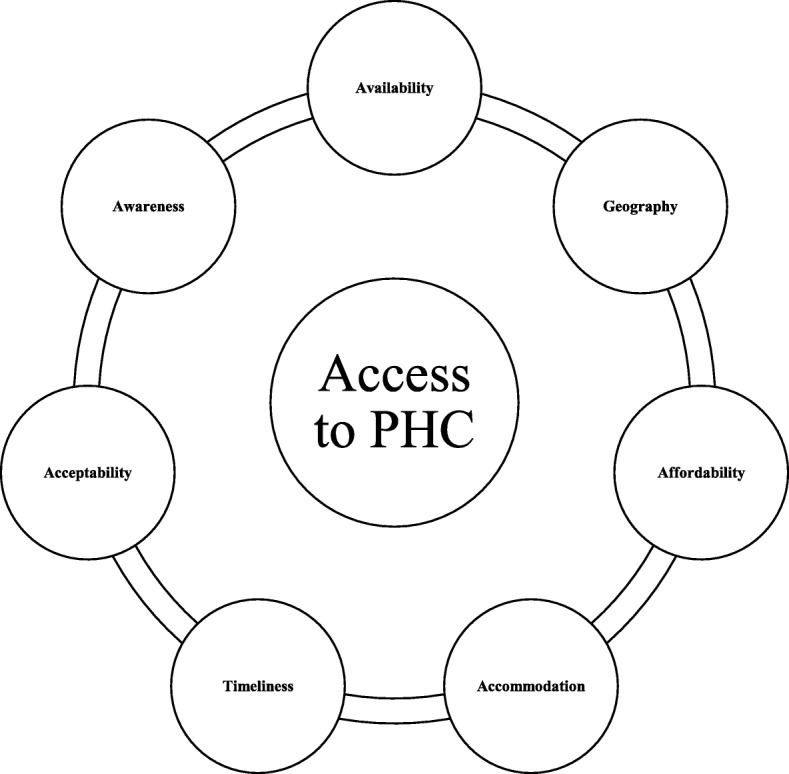
Conceptual Framework Showing Interconnections among the Access Dimensions

Many of the themes raised in this review seems to be interrelated. For instance, we found a closer relationship among availability, geography and affordability. Specifically, studies demonstrated that the absence of services in rural areas compelled clients to travel long distance in order to access health care. This travelling involves the ability to pay for transportation. Further, timely access to health care was related to affordability and availability of providers and health care facilities. Fig. 2 illustrates the interconnectedness of relationships across the dimensions.

### Discussion and Recommendations

This framework synthesis sought to understand the factors affecting health care access for PWDs in rural areas globally. We identified and mapped literature onto a rural health framework [2]. Given the number of articles found and the findings they highlight, it is evident that PWDs face many barriers in accessing PHC services in rural areas. We particularly found that PWDs were unable to access PHC due to obstacles including the interplay of four major factors; availability, acceptability, geography and affordability. For instance, limited availability of health care facilities and services and perceived low quality of care meant that those in need of health care services frequently had to travel for care. The barrier of geographic distance is worsened by transportation problems. We also observed that where health services were available most people could not afford the cost. This confirms a previous review on this issue [3] and indicates these barriers have not been resolved since the United Nations adopted the CRPD or the Sustainable Development Goals. Our synthesis also highlighted the interrelationship among the access barriers, underscoring the need to modify Russell and colleagues’ framework [2] to reflect these relationships.

Additionally, we identified similarities in access barriers for PWDs in both high-income countries and LMICs. This pattern is consistent with previous evidence which shows that access to health care is a major concern for rural populations globally, regardless of the country’s gross national income per capita [20, 21]. It is worthy to note, however, that most of the articles were based on qualitative evidence, and as a result do not provide information on the breadth of access barriers to make generalizations. Future studies should seek to conduct quantitative research about access in order to understand the barriers within a larger population of PWDs in rural areas. It would also be interesting for future studies to explore how PWDs reacted and responded to access barriers especially in resource poor settings.

The review also identified recent growing interest in disability and PHC access in LMICs. Specifically, out of the 36 retrieved articles, 26 were studies conducted in LMICs. Given this growing interest, more investment into research in other LMICs may reveal insights about the experiences of PWDs in accessing rural PHC services. It will be particularly interesting to understand this topic from health care systems with different models of governance or health care funding structures [45]. Thus, we suggest strengthening research capacity in other LMICs through appropriately targeted funding.

A prominent barrier was the inability of PWDs to afford health care. This financial barrier was due to the high cost of medical services and transportation to facilities—effectively deterring PWDs from seeking care, especially in LMICs. The finding suggests the need for governments to provide social safety nets to protect PWDs, including rolling out health insurance schemes that would ensure universal access to quality PHC services.

We also identified geography as a key feature of access to health care. In particular, our findings also indicated that PWDs in rural areas had to travel long distance to access health care. Racher and Vollman [73] have urged rural health researchers to pay attention to the characteristics of physical environment, including distance to health care facilities and services and the influence of road and weather conditions. The authors further made a clarion call for researchers to study aspects of the social environment and the political environment in relation to access because these factors are paramount to rural residents’ access to health care [73]. We found that most of the studies in the review utilized generic qualitative approaches as their study design, and adopted interviews and focus group discussions to articulate the experiences of how PWDs navigate the environment. We argue that future research could employ alternative qualitative approaches such as phenomenology and arts based methods (e.g. photographs and drawings). These approaches may provide a better understanding of key aspects of the physical, social and political environment and how they influence health care access for PWDs in rural areas in particular.

As it relates to availability as a major factor that affected clients’ access to PHC in rural areas, we revealed a general shortage of health care providers in rural areas our review. This corroborates previous reviews [3, 6]. High turnover of providers in rural areas can be expensive to health care systems and also negatively affects clients’ ability to receive quality health care [74]. For clients with disabilities, the shortage of providers in rural areas can lead to difficulties in fostering relationships and rapport that may enhance continuity of care [37, 38]. Malatzky and Bourke [75] noted that health care providers are choosing to work in urban areas despite the need and incentives to work in rural areas. They further argued that the persistent focus on workforce shortage in rural areas relative to urban areas undermines the recruitment of new health care providers to rural areas [75]. Given this, high workloads, burnouts, and restriction of opportunities for professional development and career advancement, have been documented to contribute to the notion among health care providers that working in rural areas is undesirable [6, 20, 76]. The shortage of providers may hamper efforts in achieving the 2030 Agenda for Sustainable Development Goal that reiterates equity, universality and quality of care. While attracting and retaining providers has been a major problem for rural areas globally, researchers have suggested interventions that could be effective and beneficial in guiding rural health policy and clinical practice. These include a well-defined selection criteria of students into medical training programs as well as education strategies that optimize medical training programs for rural clinical practice [77].

Furthermore, this review demonstrates that acceptability of services was a recurring theme in most of the studies. For instance, stigmatization compounded access barriers for PWDs and as a result PWDs often felt reluctant to access health care services although they may have serious health conditions that may require urgent health service intervention. Given these experiences, there is the need to factor disability issues in the design of medical education curricula, and also provide in-service training to PHC providers on how to improve their communication skills and ultimately deliver quality service to their clients with disabilities. It should be emphasized, however, that most of the studies sought the perspectives of PWDs and were fairly homogenous in highlighting negative attitudes of health care providers, particularly stigmatization and discrimination. Our findings echoes other previous literature indicating that PWDs perspectives about interactions with health care providers often cast health care providers in a bad image [57, 78]. To gain a more holistic picture of these interactions, it will be important to conduct future research to explore the perspectives of health care providers in providing care to PWDs in rural areas.

The consequences of access barriers were again revealed in the studies reviewed. Specifically, some articles in our review reported that due to the lack of health care providers and perceived quality of care in medical facilities in rural settings, some residents with disabilities and their carers resort to alternative care, including traditional and faith-based healers. Importantly, we noted that rural residents with disabilities opted for Western medical facilities after exhausting the traditional healing system. This pluralistic approach is a common health seeking behaviour of many rural residents [52]. Indeed, there have been calls on integrating traditional healing system into modern medical practices [52, 79]. However, the role of traditional and faith-based practitioners is unclear from this review. We recommend more robust research into the role of these faith-based and traditional healing systems.

Finally, the factors affecting access to PHC services for PWDs in rural areas are embedded in a complex web of different dimensions. We suggest making a change to the rural access framework in relation to health care access for PWDs. While Russell and colleagues [2] present the dimensions as independent constructs, we found interconnections among all the dimensions. In view of this, policies aimed at addressing rural access problems should consider all the dimensions and how they interact with one another rather than viewing the dimensions as distinct features.

### Limitations of the review

This review have some limitations that should be acknowledged. First, there is the possibility of not identifying all potential articles despite the systematic and transparent manner used in searching for relevant articles. This is because the main terms of this review (i.e., access to PHC, PWDs and rural) have many different interpretations and the language use around each is not yet precise. Second, the review is based on the findings reported in the various studies. As such it could be that details about the various dimensions of health care access may have been omitted due to the journals’ word limitations. Third, as we excluded peer-reviewed articles not published in English due to resource constraints, there is the possibility that we omitted relevant publications on this topic that were not published in English. Finally, publication bias may result in a wide range of studies presented in conference settings or related contexts that remain unpublished [80]; as such, there is the possibility of publication bias as we excluded grey literature. In view of these limitations, our findings may not be generalizable to rural health care access for PWDs. Nevertheless, they provide insights into rural experiences that are useful in future research, policy development and clinical practice.

## Conclusion

This review contributes to the growing body of knowledge around access to PHC for persons with disabilities in rural settings. Specifically, we illustrated how the interplay of factors such as availability, acceptability, affordability and geography affect the ability of clients with disabilities’ access to PHC services in rural settings. Importantly, we also proposed changes to Russell and colleagues’ conceptual framework [2] to capture the complex interactions of these factors in order to better conceptualized PHC access challenges and opportunities in rural settings. In view of this, we underscored the need for health policies that aimed at addressing rural access problems to consider all the dimensions of access and how they interact with one another rather than viewing the dimensions as distinct features. Finally, we identified knowledge gaps and provided recommendations for future research on this topic. In particular, we recommend more investment in research to explore the following areas in greater depth: (a) geographical contexts with health care systems different from the included studies; (b) the perspectives of health care providers; and (c) how PWDs react and respond to access barriers in rural settings, especially in resource poor settings.

## Additional file

Additional file 1: Detailed Description of the Included Articles in the Review. (DOCX 41 kb)

## Abbreviations

CRPD: Convention on the Rights of Persons with Disabilities
LMICs: Low-and Middle-Income Countries
PHC: Primary health care
PWDs: Persons with disabilities
