# Supplementary material for: Factors affecting access to primary health care services for persons with disabilities in rural areas: a “best-fit” framework synthesis
Source: Glob Health Res Policy. 2018 Dec 25;3:36. doi: 10.1186/s41256-018-0091-x (PMC6305566; doi:10.1186/s41256-018-0091-x)
Supplement: Supplementary file 1 — Detailed Description of the Included Articles in the Review. (DOCX 41 kb) [file 41256_2018_91_MOESM1_ESM.docx]

**Additional File 1: Detailed Description of the Included Articles in the Review**

| **No** | **Author, Year and Country of Publication** | **Study Aim and Design** | **Study Population** | **Data Collection and Analysis** | **Main Findings Related to Access to Primary Health Care Services in Rural Areas** |
| --- | --- | --- | --- | --- | --- |
| 1 | Ahmad, 2013 (Pakistan) | **Objective**: To assess persons with physical disabilities access to health care with particular emphasis on the barriers of access  **Design**: Qualitative study | Different participants  —245 persons with physical disabilities (171 males and 74 females) aged 15-35 years  —district health managers  —1 director in charge of public buildings | **Data collection:** In-depth interviews  **Data analysis:**  Thematic analysis | **Acceptability:** clients faced discrimination in receiving health care  **Accommodation:** architectural designs of buildings and transport service are not disability friendly  **Affordability:** high cost of health service and transportation  **Availability:** lack of health care providers and services  **Awareness:** health care providers lack of knowledge on disability health needs  **Timeliness:** long queues resulting in clients delay in receiving health care |
| 2 | Asher et al., 2017 (Ethiopia) | **Objective:** To understand the experiences and reasons for the restraint of people with schizophrenia  **Design:** Qualitative study | 50 participants (24 males and 26 females) aged 25 years and above  —4 people with schizophrenia  —17 caregivers  —7 community leaders  —22 key informants | **Data collection:** In-depth interviews and focus group discussions  **Data analysis:** Thematic analysis | **Affordability:** Lack of affordable treatment options  **Availability:** absence of mental health care service  **Geography:** long distance in reaching to services |
| 3 | Banks et al., 2016 (Ethiopia) | **Objective**: To examine explanatory models, health-seeking behaviors and self-care amongst persons with podoconiosis  **Design**: Qualitative study | 34 participants (19 males and 15 females) aged 26-90 years  —27 persons with podoconiosis (13 males and 14 females)  —5 patient association leaders (4 males and 1 female)  —2 health care providers ( males). | **Data collection:** In-depth interviews, focus group discussions and key informant interviews  **Data analysis** Thematic analysis | **Acceptability:** clients’ lack of trust in modern medications, only use medical clinics as last resort  **Awareness:** clients’ lack of knowledge about modern medications |
| 4 | Braathen et al., 2013 (South Africa) | **Objective:** To elucidate core issues that are key to the development of global mental health agenda  **Design:** Qualitative grounded theory approach | Different participants  —1 woman (aged 65years) with a mental disorder  —Family members of the person with mental disorder | **Data collection:** Observation, in-depth interviews, contextual data from various health sectors  **Data analysis:** Grounded theory analysis | **Affordability:** high cost of transportation to a facility  **Availability:** shortage of health care providers, equipment and medication  **Awareness:** health literacy relating to the lack of family and community understanding of mental disorder  **Geography:** Clients trek long distance with public transport to a facility. The area is characterized by geographical features including rugged hills, deep valleys, rivers and forests.  **Timeliness:** shortage of health care providers culminates in little time for health delivery to clients |
| 5 | Burton and Walters, 2013 (Australia) | **Objective:** To explore people with Intellectual Disabilities (ID) access to an annual comprehensive health assessments (ACHAs) and factors that affect their access to ACHAs  **Design:** Qualitative approach | 18 participants  —8 adults with intellectual disabilities  —6 caregivers/support workers of persons with persons with intellectual disabilities  —4 health care providers | **Data collection:** Semi-structured interviews  **Data analysis:** Thematic analysis | **Awareness:** clients and carers lack of knowledge about health assessments. Health care providers’ also have limitations in disability health issues. Communication triangle among health care providers, clients and carers as advocates/interpreters is critical to effective health care delivery.  **Timeliness:** clients often have to wait for a long period to see a preferred health care provider |
| 6 | Davidsson and Södergård, 2016  (USA) | **Objective:** To explore the perceptions about factors that promotes and impedes health care access for persons with physical disabilities in rural Louisiana  **Design:** Qualitative approach | 9 persons with physical disabilities (5 females and 4 males) aged 46-87 years | **Data collection:** In-depth interviews  **Data analysis:** Content analysis | **Acceptability:** clients expressed trust and confidence in health care providers’ knowledge about disability issues.  **Accommodation:** designs of medical equipment and the doctor’s office are not disability friendly  **Affordability:** clients have health insurance coverage, however the ability to pay for health care depends on insurance type  **Awareness:** one client got health care information via television or other people, while others got it from health care providers’ especially booking appointments.  **Geography:** clients were all dependent on transportation to get to health care providers |
| 7 | Di Blasi et al., 2006 (Australia) | **Objectives: (1)** To gain an understanding of pharmacists’ perspectives of and barriers to the provision of health  care services to persons with intellectual disabilities (ID); and **(2)** To explore strategies and solutions for improving pharmacists’ involvement in delivering health care services to persons with ID  **Design:** Descriptive qualitative study | 10 Community pharmacists (5 males and females each) aged 23-58 years and with 6 months to 37 years’ experience as pharmacists. | **Data collection:** Semi-structured interviews  **Data analysis:** Thematic analysis | **Acceptability:** health care providers ensured medication safety and also built a strong relationship/rapport with clients (persons with intellectual disabilities).  **Availability:** staff shortage hampered health care delivery to clients  **Awareness:** cognitive abilities and capacity of clients to understand their medications and health care providers to effectively communicate to clients  **Timeliness:** insufficient time to deliver service to clients |
| 8 | Eide et al., 2015 (South Africa, Namibia, Malawi and Sudan) | **Objective:** To both identify the magnitude and specific barriers, and also estimate the impact of disability on health care access barriers  **Design:** Quantitative study | 9307 individuals with and without disabilities in 400-500 households in 4 countries —South Africa, Namibia, Malawi and Sudan | **Data collection:** Survey questionnaires  **Data analysis:** Structural equation modelling | **Affordability:** high cost health care services  **Availability:** shortage of health care services and equipment  **Geography:** Lack of transport, long distance and unfavorable terrain to health care facilities |
| 9 | Goodridge et al., 2015 (Canada) | **Objective:** To examine the perspectives of persons living with traumatic spinal cord injuries (tSCI) on their access to health care and support services  **Study design:** Qualitative descriptive social phenomenology | 23 adults living with tSCI (16 males and 7 females) aged 18 and over.  —10 participants (9 men, 1 woman) reside in rural areas.  —13 participants (7 men and 6 women) reside in urban centers. | **Data collection:** Semi-structured interviews  **Data analysis:** Thematic analysis | **Acceptability:** clients described both positive and negative attitudes of health care providers  **Accommodation:** access to health care facilities was a challenge to clients. Health care providers were willing to be flexible with bureaucratic procedures  **Geography:** some clients could drive and this facilitated their access to health care services  **Timeliness:** timely access to care posed a challenge to clients |
| 10 | Grut et al., 2012  (South Africa) | **Objective:** To explore the difficulties persons living in a resource-poor environment access and utilize health care services, and how this may render inaccessible health care services when they are available  **Design:** Qualitative study | Different participants  —24 persons with disabilities and/or their family members (14 men and boys, and 10 women and girls)  —18 health care providers (13 health care professional and 5 unskilled workers at a health facility) | **Data collection:** In-depth interviews  **Data analysis:** Contextual and interpretive analysis | **Acceptability:** past experience increases carers expectations about health care quality to clients  **Affordability:** extra transport cost for accompanied carer and wheelchair. Some clients do not have disability grant as income source for health care expenses  **Geography:** some carers use public transport to take the client to a health facility, but has to resort to wheelbarrow because the client becomes violent when in public transport. |
| 11 | Grut et al., 2015 (Malawi) | **Objective:** To present a understanding of access to tuberculosis (TB) services for people with disabilities (PWDs) in a resource poor context  **Design:** Qualitative approach | 89 participants (53 males and 36 females)  —47 PWDs-physical sensory, intellectual and albinism (23 males and 24 females)  —11 parents/guardians ( 1 male and 10 females)  —31 key informants | **Data collection:** Semi-structured interviews and site observations  **Data analysis:** Thematic analysis | **Acceptability:** health care providers treated clients with respect and also prioritize them in queues. This often results in anger from other patients  **Availability:** health care facility lacks equipment and personnel for TB testing and medication  **Awareness:** clients lack of knowledge about TB causes and medications  **Geography:** some participants could not transport themselves to health care facility |
| 12 | Hailemariam et al., 2017 (Ethiopia) | **Objective:**  To explored barriers to initial and ongoing engagement of people with severe mental disorders  **Design:** Qualitative phenomenological approach | 70 participants (31 males and 39 females) aged 25 years and above  —20 health extension workers  —11 primary health care staff  —22 caregivers  —17 people with severe mental disorders | **Data collection:** In-depth interviews and focus group discussion  **Data analysis:** Thematic analysis | **Acceptability:** lack of trust in modern medication  **Affordability:** clients unable to afford cost of care due to poverty  **Availability:** limited medication options to clients  **Geography:** long distance in getting to a facility |
| 13 | Hailemariam et al., 2016 (Ethiopia) | **Objective:** To inform delivery of a new primary care-based mental health service through the identification of barriers to equitable access to mental health care and strategies of overcoming the barriers.  **Design:** Qualitative study | 33 participants (16 males and 17 females) aged 25-65 years  —12 health extension workers  —6 service users  —5 caregivers  —3 traditional and faith healers  —4 community leaders  —2 NGO representatives  —1 health office representative | **Data collection:** In-depth interviews and a focus group discussion  **Data analysis:** Framework analysis | **Acceptability:** lack of trust in modern medication, however those who received care at mental health facilities can wipe out the fear of modern medicine.  **Affordability:** clients with sufficient financial pay for care, transportation, accommodation and meal  **Availability:** absence of mental health care service in the district  **Awareness:** community lacks awareness about primary mental health care  **Geography:** long distance in reaching to mental health services  **Timeliness:** lack of mental health services in the community hampered timely access to care |
| 14 | Hamilton et al., 2017 (United States) | **Objective:** To describe the utilization, accessibility, and satisfaction of health care services for persons with spinal cord injuries  **Design:** Quantitative study | 142 participants (50 females, 92 males) living with spinal cord injuries | **Data collection:** Survey questionnaires  **Data analysis:** Descriptive statistics, Wilcoxon rank-sum tests and Spearman correlations | **Acceptability:** clients expressed satisfaction with the care they have received  **Accommodation:** clients reported accessibility issues both inside and outside facilities (e.g. washrooms, doorways, exam tables and rooms, labs, imaging and parking spaces)  **Awareness:** Majority of clients indicated physician knowledge and good communication about their health care needs |
| 15 | Hanlon et al., 2017 (Ethiopia) | **Objective:** To explore the barriers, facilitators and potential strategies to promote good health system governance with respect to mental health care in Ethiopia  **Design:** Qualitative study | 17 participants  —7 national/regional level policy makers, planners and service developers makers  —10 district level health office administrators and facility heads. | **Data collection:** Semi-structured interviews  **Data analysis:** Framework analysis | **Acceptability:** people are reluctant to accepting biomedical approaches to mental health care. Stigma is also attached to mental health care  **Affordability:** Medications can be expensive but integrating mental care into PHC will reduce the cost. Policies like insurance and poverty reduction strategies will promote equitable access.  **Availability:** lack of medication supplies and personnel  **Awareness:** low level of mental health awareness among policy makers and community members  **Geography:** clients can receive care when it is closer  **Timeliness:** timely intervention of care can lead to better illness outcomes. |
| 16 | Hussain and Tait, 2015 (Australia) | **Objective:** To describe the perceptions and experiences of primary caregivers on information needs, perceptions about service providers and experiences of rural service provision  **Study design:** Qualitative study | 17 parents (1 male and 16 females) of children with developmental disabilities aged 23-59 years | **Data collection:** Face-to-face in-depth interviews  **Data analysis:** Thematic analysis | **Acceptability:** parents expressed negative attitudes of health care providers  **Availability:** lack of experienced health care providers and limited health services  **Awareness:** lack of adequate and timely information about health services awareness among community members  **Geography:** parents had to drive long distance for care  **Timeliness:** long waiting list to get care in the community |
| 17 | Iezzoni et al., 2006 (United States) | **Objective:** To explore the experiences of rural residents with disabilities access to health  care  **Design:** Qualitative  study | 35 adults with sensory,  physical and mental disabilities (15 males and 20 females) aged 21-64 years | **Data collection:** Focus group interviews  **Data analysis:** Thematic analysis | **Acceptability:** clients underscored their personal relationship with health care providers  **Accommodation:** facilities, equipment and transportation are not disability friendly  **Availability:** limited health services and providers  **Awareness:** health care providers lack of knowledge about disability health issues and clients limited knowledge in health issue  **Affordability:** clients have insurance but are unable to pay for medications not covered by their insurance.  **Geography:** clients had to travel for care  **Timeliness:** transportation problems leads to delays in receiving care that is not available locally |
| 18 | Järnhammer et al., 2017 (Nepal) | **Objective:** To explore experiences of persons using lower-limb prostheses, in relation to the Convention on the Rights of Persons with Disabilities articles on mobility, education, health, rehabilitation, and work and employment  **Design:** Qualitative study | 16 persons using lower limb prostheses (10 males and 6 females) aged 21-67 years  —9 (5 males and 4 females) reside in rural areas  —7 (5 males and 2 females) reside in urban areas) | **Data collection:** Individual interviews  **Data analysis:** Content  analysis | **Acceptability:** clients expressed satisfaction with the health care they received**.** They also highlighted positive attitudes of health care providers  **Affordability:** cost of service is low at health facilities. Clients however paid for health expenses through loans or relatives.  **Geography:** health post is closer to participants but hospital is within bus distance |
| 19 | Knox et al., 2014 (Canada) | **Objective:** To explore health care provider perspectives on persons with traumatic spinal cord injuries access to care  **Design:** Qualitative study | 23 health care providers (therapists, physicians, nurses, home care workers and managers)  —9 provide services in rural communities  —14 work in urban centers | **Data collection:** Semi-structured interviews and focus groups  **Data analysis:** Framework analysis | **Accommodation:** while facilities are wheelchair accessible getting around most sidewalks is difficult  **Affordability:** existence of inequalities and inequities of affordable health services  **Geography:** transportation for health care services posed a challenge to clients  **Awareness:** easy communication among health care providers culminated in smooth health care delivery |
| 20 | Loyola-Sanchez et al., 2016 (Mexico) | **Objective:** To describe the impact of people living with arthritis, identify their perceived health needs and understand the barriers they encounter in accessing health care services  **Study design:** Qualitative ethnography | 65 participants  —32 people living with arthritis (14 males and 17 females) aged 30-85 years  —19 public health care providers  —4 traditional health providers  —4 family members  —5 community leaders  —1 municipal authority | **Data collection:** interviews, fieldwork observations and community meetings  **Data analysis:** Framework  approach | **Acceptability:** ethnic discrimination from health care providers hampered clients’ access to care  **Affordability:** clients lack of financial resources made them not to seek care  **Availability:** absence of health care services in the municipality  **Awareness:** clients lack information about the health care system. Providers also lack knowledge on disability health issues  **Geography:** clients had to travel long distance for services but there is inefficient transportation |
| 21 | Mji et al., 2017 (South Africa) | **Objective:** To explore how activity limitations interact with factors related to context, systems, community and personal factors in accessing public health care  **Design:** Qualitative study based on different case studies | 4 persons living with disabilities  —2 persons with physical disabilities  —2 persons with sensory impairments | **Data collection:** In-depth interviews  **Data analysis:** Thematic analysis | **Acceptability:** clients highlighted positive attitudes of health care providers  **Accommodation:** health facility operates on certain hours and/or days of the week  **Affordability:** high cost of care and transportation  **Availability:** lack of health care providers and services  **Awareness:** health care providers had difficulties in communicating to a client with hearing impairment  **Geography:** clients had to travel vast distances for care  **Timeliness:** clients encountered long waiting times at health facility |
| 22 | Mshana et al., 2011 (Tanzania) | **Objective:** To investigate the experience and treatment seeking behaviors of persons with Parkinson disease, their carers and community understandings of the disease  **Design:** Qualitative study | 62 participants (32  males and 30 females)  —28 persons with Parkinson disease aged 45-94 years  —28 carers  —4 health workers  —2 traditional healers | **Data collection:** Semi-structured interviews and focus group discussions  **Data analysis:** Inductive approach | **Affordability:** clients received financial support from family members to purchase drugs and for transportation to and from hospital  **Availability:** drugs are not available in health facilities or local pharmacies |
| 23 | Ngo et al., 2013 (Vietnam) | **Objective:** To examine the experiences of young people with disabilities from ethnic minorities and their families.  **Design:** Qualitative participatory study | 55 participants  —24 children and youth with physical and/or sensory disabilities (15 males and 9 females) aged 14-19 years  —31 mothers of a child/children with disabilities | **Data collection:** In-depth interviews and focus group discussions  **Data analysis:** Thematic analysis | **Acceptability:** clients experienced marginalization by health care providers  **Affordability:** parents could not afford medical cost and other indirect cost like accommodation and meals. As a result they took loan for these expenses  **Awareness:** parents lack of awareness of some services like free health-checks and medication  **Geography:** parents had to rely on motorbike to health facilities with their children with disabilities |
| 24 | Nualnetr and Sakhornkhan, 2012 (Thailand) | **Objective:** To develop an action plan in order to improve access to home health care and assistive devices for persons with disabilities and evaluate changes in the numbers for those who received such services  **Design:** Mixed method | Different participants  —99 persons with disabilities (42 females and 57 males)  —village health volunteers  —primary health care staff  —community leaders and members  —caregivers  —administrative officers | **Data collection:** Group meetings and interviews  **Data analysis:** Descriptive statistics | **Geography:** health care providers could not provide home health care to their clients due to the terrain, especially during the period of heavy rains and floods. |
| 25 | Reddy et al., 2014 (India) | **Objective:** To explore factors that prevent schizophrenia patients and their families’ access to psychiatric treatment.  **Design:** Qualitative study | 16 family members of persons with schizophrenia (9 males and 7 females) | **Data collection:** In-depth  interviews  **Data analysis:** Unclear | **Affordability:** family members inability to raise funds to treat persons with schizophrenia  **Geography**: difficulties in reaching to health care facility due to bad road network |
| 26 | Tilahun et al., 2017 (Ethiopia) | **Objective:** To examine training needs and perspectives of community health extension workers in relation to providing child mental health care  **Design:** mixed methods approach (cross-sectional  survey and qualitative study) | 104 health extension workers (all females) | **Data collection:**  —Quantitative (structured questionnaire)  —Qualitative (11 in-depth interviews)  **Data analysis:**  —Quantitative (descriptive analysis)  —Qualitative (framework analysis) | **Acceptability:** Negative attitudes from community towards persons with mental problems  **Affordability:** families face financial problems for treatment and transportation to access mental health services  **Availability:** lack of skilled professionals and treatment at primary health care settings  **Awareness:** health care providers lack of knowledge about mental health problems |
| 27 | Tora et al., 2012 (Ethiopia) | **Objective:** To explore factors related to discontinued attendance at treatment and prevention clinics by patients with podoconiosis  **Design:** Qualitative study | 88 participants (40 males and 48 females) aged 16-75 years.  —44 persons with podoconiosis  —policy makers  —community and religious leaders  —health agents and social workers. | **Data collection:** In-depth interviews, focus group discussions and key informant interviews  **Data analysis** Thematic analysis | **Acceptability:** stigma deterred clients from seeking treatment. Clients also doubted the quality of treatment  **Affordability**: clients are unable to transport although the treatment was free  **Geography:** clinic sites were remotely from the clients. The clients encountered difficulties in finding transport and always walked on long distance to the health facilities |
| 28 | Tsegay et al., 2015 (Ethiopia) | **Objective:** To explore barriers to access and re-attendance of persons with podoconiosis  **Design:** Qualitative study | 53 participants aged 20-65 years.  —36 persons with podoconiosis (17 males and 19 females)  —8 patient association leaders (6 males and 2 females)  —9 health care providers (6 males and 3 females). | **Data collection:** In-depth interviews and focus group discussions  **Data analysis:** Thematic analysis | **Acceptability:** anxiety over stigma deterred patients from seeking treatment  **Affordability:** clients did not want to abandon their commitments in economic activities and seek care. Clients could not also afford transportation and accommodation  **Awareness:** community’s awareness about the condition (podoconiosis) and treatment is generally low  **Geography:** clients walked long distance on foot (at times over mountains and rivers) to seek treatment |
| 29 | Van Hees et al., 2014 (Nepal) | **Objective:** To gain a better understanding of the perceived barriers faced by persons with disabilities in accessing primary health care services  **Design:** Qualitative study | 21 participants  — 9 health care providers  —11 persons with disabilities (physically or sensory impairments)  —1 person from Disabled People’s Organization | **Data collection:** Semi-structured interviews  **Data analysis:** Analytical induction | **Acceptability:** Clients’ self-stigma prevented them from seeking care. Clients also prefer care at medical shops  **Accommodation:** architectural designs of health facilities are not disability friendly  **Affordability:** poor financial conditions of clients due to lack of employment  **Availability:** lack of staff and services  **Awareness:** health care providers lack of knowledge about disability issues and also difficulties in communicating to clients with hearing impairments.  **Geography:** clients noted distance to facility and bad nature of roads. Public transportation is free for clients |
| 30 | Van Rooy et al., 2012 (Namibia) | **Objective:** To investigate the experiences of people living with disabilities access health care facilities and services  **Design:** Qualitative study | 25 persons with disabilities, however table 1 ( page 767) has participants characteristics which shows that 206 persons (84 males and 122 females) participated | **Data collection:** Semi-structured interviews  **Data analysis:** Thematic analysis | **Acceptability:** clients expressed both positive and negative attitudes of health care providers  **Accommodation:** health facilities operate on daily basis  **Affordability:** cost of transportation and services  **Availability:** lack of providers and services in facilities  **Awareness:** lack of interpreters for clients with hearing impairments. Language differences impeded communication between providers and clients  **Geography:** clients used different modes of transportation to health facility  **Timeliness:** clients experienced long queues at health facilities |
| 31 | Varghese et al., 2015 (India) | **Objective:** To identify health seeking behaviors of families with children with intellectual disabilities and the barriers they faced accessing health care  **Design:** Qualitative study | 15 participants  —10 caregivers  —5 health care providers | **Data collection:** Semi-structured interviews and focus group discussions  **Data analysis:** Thematic analysis | **Acceptability:** Negative and positive attitudes of health care providers towards children with intellectual disabilities  **Affordability:** caregivers had to sell their wheat, land or businesses in order to raise money for the child’s treatment  **Availability:** presence of health care providers  **Awareness**: inadequate information about treatment options for the children with intellectual disabilities  **Geography**: caregivers raised transportation difficulties |
| 32 | Vergunst et al., 2017 (South Africa) | **Objective:** To explore the issue of access to health care services for persons with disabilities in rural South Africa  **Design:** Quantitative observational cross-sectional study | 773 participants aged 5-97 years covering 527 households  —322 persons with disability  —451 controls (without disability) | **Data collection:** Interviews  **Data analysis:** Descriptive and inferential statistics | **Acceptability:** clients encounter negative attitudes of providers  **Accommodation:** designs of facilities are not disability friendly  **Affordability:** clients could not afford cost of transportation and care  **Availability:** inadequate medical equipment and services  **Awareness:** clients lack of knowledge about services and also difficulties in communicating with providers  **Geography:** lack of accessible transport to health facility |
| 33 | Vergunst et al., 2015 (South Africa) | **Objective:** To explore the challenges faced by people with disabilities in accessing health care  **Design:** Qualitative study | 26 participants (8 males and 18 females) aged 5years and over  —9 persons with disabilities (5 physical, 2 mental, 1 sensory and 1 cognitive and physical  Impairments)  —9 health care providers  —8 community Members (chiefs and traditional leaders) | **Data collection:** Semi-structured interviews  **Data analysis:** Thematic content analysis | **Acceptability:** stigma mostly from health care providers impeded health access.  **Accommodation:** designs of transport and health facilities are not disability friendly  **Affordability:** high cost of transportation to health facility  **Availability:** inadequate medical equipment and services  **Geography:** clients had to travel long distance to a facility  **Timeliness:** an average wait period is half a day to access health care |
| 34 | Walker et al., 2016 (USA) | **Objective:** To examine barriers and facilitators to accessing health and support services among urban and rural families of children with disabilities  **Design:** Qualitative study | 19 parents of adolescents living with disabilities (physical, mental, intellectual, sensory and multiple disabilities)  —9 parents/guardians living in rural areas  —10 parents living in urban areas | **Data collection:** In-depth interviews  **Data analysis:** Content analysis | **Affordability:** high cost of service not covered by insurance  **Availability:** limited availability of quality service **Geography:** limited public transportation. This resulted in long travel time and wait time for transport |
| 35 | Wark et al., 2015 (Australia) | **Objective:** To undertake an exploration of the experiences of individuals ageing with a learning disability in rural areas.  **Design:** Qualitative study | 34 participants  —17 older adults with a learning disability (10 males and 7 females) aged 54-79 years.  —17 carers (3 males and 14 females) | **Data collection:** Semi-structured in-depth interviews  **Data analysis:** Thematic analysis | **Acceptability:** support workers expressed positive attitudes of health care providers  **Accommodation:** difficulties in clients getting medical appointment  **Availability:** lack of medical services  **Geography:** ‘tranny of distance’ posed a major challenge to health care access  **Timeliness:** considerable wait time to see a doctor |
| 36 | Wongkongdech and Laohasiriwong, 2014 (Thailand) | **Objective:** To explore accessibility to health services among persons with mobility disabilities, and factors influencing such access  **Design:** Quantitative-Cross sectional study | —462 persons with physical disability (253 males and 209 females)  —324 reside in rural areas  —138 reside in urban areas | **Data collection:** Interviews  **Data analysis:** Descriptive statistics and multiple regression analysis | **Acceptability:** clients expressed better quality of care at health care facilities  **Accommodation:** clients indicated wait time to receive care  **Affordability**: clients with low income less likely to afford health care  **Availability:** clients highlighted adequacy of care  **Awareness:** lack of knowledge about their rights to health care  **Geography:**  Clients’ transportation mode to a health care facility: wheelchairs (57.36%) and walking (32.90%). |
